# A focus on penetration index – a new descriptor of chemical bonding

**DOI:** 10.1039/d3sc90191b

**Published:** 2023-10-13

**Authors:** Wojciech Grochala

**Affiliations:** a Center of New Technologies, University of Warsaw Zwirki i Wigury 93 02-089 Warsaw Poland w.grochala@cent.uw.edu.pl

## Abstract

Electron clouds surrounding atoms interpenetrate in a molecule, due to weak van der Waals interactions or formation of a genuine chemical bond. Now, Alvarez and Echeverría (S. Alvarez, J. Echeverría, *Chem. Sci.*, 2023, https://doi.org/10.1039/D3SC02238B) suggest a simple descriptor of how deep this interpenetration is, calling it a *penetration index*, *i* [Å]. This property may easily be related to a combined thickness of the van der Waals regions of two bond-forming atoms, thus giving rise to a *dimensionless penetration index*, *p*_AB_ [%]. How far this new index may take us will be discussed in this article.

## Abstract 2

Electron clouds surrounding atoms interpenetrate in a molecule, due to weak van der Waals interactions or formation of a genuine chemical bond. Now, Alvarez and Echeverría have suggested a simple descriptor of how deep this interpenetration is, and called it a *penetration index*, *i*.

## Graphical abstract

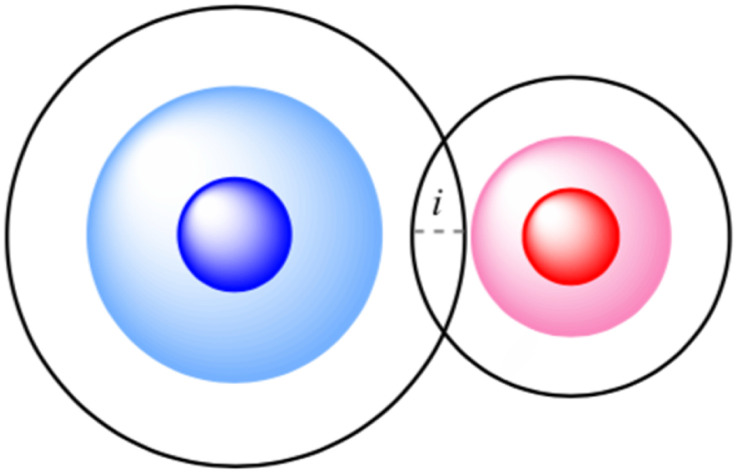

“*I believe the chemical bond is not so simple as some people seem to think*” – this famous quote from prime theoretician of his time, Robert Mulliken, says a lot.^1^ After three quarters of a century, Roald Hoffmann, over forty years older than his predecessor-in-science, reaffirms: “*There is nothing more fundamental to chemistry than the chemical bond. But it’s not a simple concept*”.^2^ And he says so despite the fact that enormous progress has been achieved in understanding chemical bonds of all flavors during the decades that passed since Lewis first drew, in 1902, his “cubical atoms”.^3^

The concept of “cubical atoms” by Gilbert N. Lewis, one of the founding fathers of the modern chemical bonding ideas,^3^ is now taught as the “octet rule”^4^ (or the “doublet rule” for H, He and Li^+^-like species). Despite its limitations, but thanks to its enormous simplicity, this approach has been used to qualitatively rationalize chemical bonding both in isolated molecules and in the solid state (*e.g. via* Zintl–Klemm rules).^5,6^ It is hard to underestimate its importance for didactics of chemistry – counting to eight is hardly an obstacle for any pupil.

More complex ideas soon came with further advances in quantum chemistry. One of Pauling’s rules, for example, asserted that of two orbitals in an atom, the one that could overlap the most with an orbital from another atom would form the strongest bond.^7^ In this way, introducing overlap integrals, more features could be explained in a semiquantitative way, including the bond strengths of diverse chemical bonds. An even more quantitative approach was offered by Badger in 1934, who noticed that a simple relationship exists between internuclear distances and bond force constants.^8^ This rule, which was revisited numerous times by our community,^9^ opened a way to constructive dialogue between specialists in bond-length determination (*e.g. via* X-ray, neutron or electron diffraction), spectroscopists (vibrational spectra and bond dissociation energy determinations) and quantum chemists. The deviations from this rule are rare and therefore very interesting.

Somewhat more complex situations are encountered in extended solids, particularly in three-dimensional coordination polymers, or in elemental metals and alloys. Here, unlike in most molecules, an atom may have a very complex bonding environment with several (usually up to a dozen) atoms found in its first coordination sphere, at different interatomic separations, and with some secondary bonding too. It is impossible, in most cases, even in a *Gedankenexperiment*, or thought experiment, to dissociate only one of these bonds completely, leaving all others intact (and therefore to determine dissociation energy of one particular bond). Equally, separating a “localized oscillator” from all others is virtually impossible. Clearly, the “metallic state” gave trouble to Pauling, who departed from using integer numbers, and came up with an oversimplified and yet fuzzy description of chemical bonding in metals, including the simultaneous presence of M^0^, M^+^ and M^−^ species in the crystal lattice in a peculiar proportion.^10^ In consequence, other descriptors of chemical bond strength have been used in such cases, including lattice energy (related to dissociation into isolated ions in the gas phase) or cohesive energy (dissociation into atoms in the gas phase). But these “collective properties” hardly characterize an isolated bond within a solid. Therefore, the precise distribution of electronic density, which is these days accessible from X-ray diffraction, and the associated theoretical framework of *atoms-in-molecules* by Bader that permits separation of density into atomic basins,^11^ serves as a utile tool to characterize patterns of chemical bonding in extended solids. Many more useful (often quite complex) theoretical descriptors of chemical bonding appeared, and it is not possible to mention them all in this short commentary.

Bond length is a primary descriptor of any chemical bonding, and it is used to describe solids as well. A chemist’s typical way of thinking is that if a separation between two atoms in the solid state exceeds the sum of the van der Waals (vdW) radii of these atoms, then there is no appreciable interaction between them. If the said separation is shorter than the respective sum, then there is some interaction. If the separation is very short, and approaches the sum of atomic or ionic radii of these elements, then there is a genuine chemical bond between them. The closer atoms get together (non-aggravated by external pressure), the stronger the chemical bond.

Now, Alvarez and Echeverría^12^ suggest looking at chemical bonding in isolated molecules or in extended solids using a new quantitative descriptor. This new index is, in some ways, related to the size of the electronic cloud surrounding the atom, and certainly reflects the way of thinking along the lines described in the preceding paragraph. They define the penetration index between atoms A and B as:

1 *i*_AB_ = *v*_A_ + *v*_B_ − *d*_AB_

where *v*_x_ is the vdW radius of element x (x = A, B), while *d*_AB_ is the distance between the nuclei of the A and B elements. Then, they convert this into a dimensionless descriptor:

2 *p*_AB_ = 100·(*v*_A_ + *v*_B_ − *d*_AB_)/[(*v*_A_ − *r*_A_) + (*v*_B_ − *r*_B_)] [%]

where the corresponding *r*_i_ corresponds to the *covalent radius* of element i.

In this way, *p*_AB_ measures the degree to which *i*_AB_ penetrates the combined width of the vdW regions of two atoms, A and B. The benefit from such definition is that *p*_AB_ = 0% corresponds to the situation when the two vdW spheres are just touching each other (*i.e.*, *d*_AB_ = *v*_A_ + *v*_B_), while *p*_AB_ = 100% means they are interpenetrating up to the point where the two valence spheres touch each other (*i.e.*, *d*_AB_ = *r*_A_ + *r*_B_). The first instance corresponds formally to no bonding at all, the latter to a “classical covalent bonding”.

These authors have analyzed an impressive number of systems using their new descriptor and they confirmed its usefulness for describing all sorts of interatomic interactions, from very weak to very strong. An emerging picture, that there is a *continuity of bonding* across the entire vdW crust all the way to the valence region, nicely confirms what has already been seen in numerous experiments (particularly those at elevated external pressure^13^). The summarizing Fig. 46 in the original article (reproduced herein as Fig. 1) shows that the new index, *p*_AB_, beautifully describes chemical bonding across diverse systems and bonding situations.

**Fig. 1:**
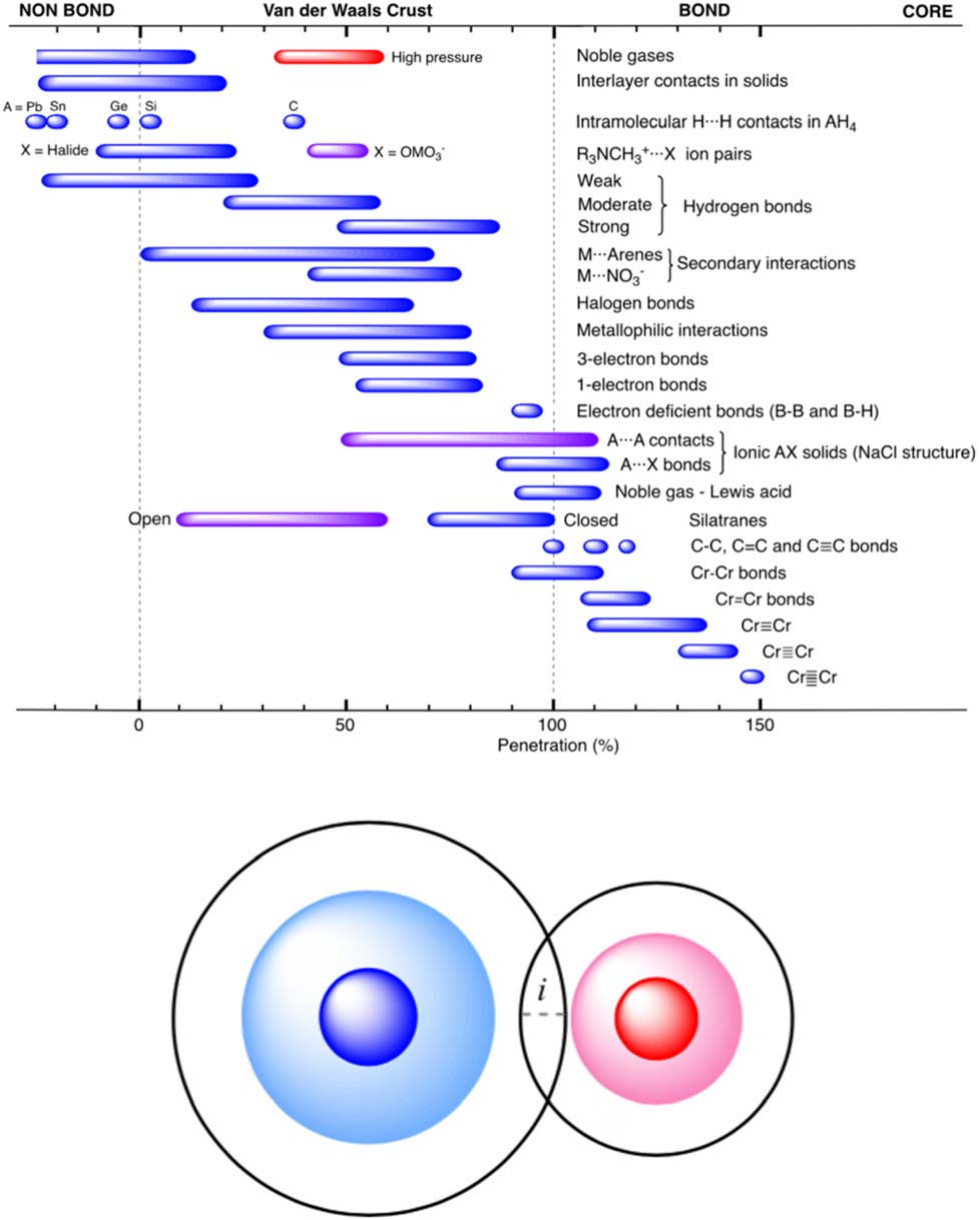
Ranges of penetration indices presented by the different bond types discussed in ref. 12, showing the continuous distribution from the very low penetration of the pure van der Waals interactions to the strongly covalent metal–metal quintuple bonds. Reproduced from ref. 12, with permission from the Royal Society of Chemistry.

This unified picture holds from the region of very low penetration (pure van der Waals interactions) to the strongly covalent and very short metal–metal quintuple bonds, where the penetration index approaches 150%. It describes very nicely the complex nature of weak 1- and 3-electron bonds, halogen bonds and hydrogen bonds, as well as donor–acceptor (acid/base) interactions.

The *p*_AB_ is certainly a didactically useful index, just like, *e.g.*, bond order; the explanatory strength of both integers and percent values is that they are simple to convey and thus are useful pedagogical tools for those teaching introductory chemistry. However, it should be stressed that – despite what Fig. 1 seems to suggest – its applicability seems to be rather qualitative, and one should use *p*_AB_ with great caution when comparing two vaguely related bonds and sometimes even the related ones.

## The problem of very different relative thicknesses of the vdW crusts

**Fig. 2:**
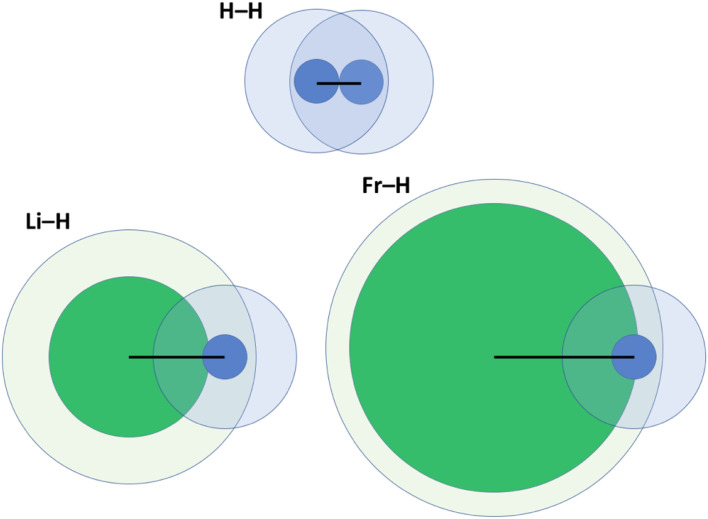
Illustration of penetration of the vdW crust and valence region for three related molecules: H_2_, LiH and FrH.^14,15^ Here, green fields belong to alkali metals and blue ones to H. Inner cores represent the valence region and more diffuse ones the vdW region. Black lines show the bond length in a diatomic molecule.

One key problem with the definition according to eqn (2) is that the size of the vdW crust relative to the valence region (*V*_i_/*R*_i_) is not identical for all elements. Indeed, this may greatly vary; for example, *V*_i_/*R*_i_ is *ca.* 4.47 for He, and as little as 1.17 for Fr.^14^ In other words, the vdW crust is very thick relative to the valence region for the former, and very thin for the latter. At first, this does not seem to be a problem, as *p*_AB_ may of course be defined for any combination of two atoms. However, upon closer inspection we do detect a problem, which is illustrated in Fig. 2 and described below.

For H_2_, the *p*_AB_ takes the value of 99.9%. It takes a very similar value of 100% for Li_2_. In these cases, the valence spheres touch each other, and one may expect the presence of a covalent bond (a single sigma bond in both cases). On the other hand, for a LiH molecule (with its partly ionic character), the *p*_AB_ equals 107.1%. This is not extremely far from those for H_2_ and Li_2_. In any case, this index tells us qualitatively that there is slight penetration of the valence regions aside from the vdW ones. In the extreme case of a FrH molecule, the *p*_AB_ equals 135%, and thus it is the largest among the four. Given what Fig. 1 seems to suggest, one would expect the strongest chemical bonding in this series to be seen for FrH, while the factual trend is just the opposite (the dissociation energies are vastly different: 4.5 eV for H_2_, 2.5 eV for LiH, and 1.7 eV for FrH).

One might expect (even given the complexity of the electronic structure of Fr and the increasing (p,d,f)-orbital character of its valence orbitals) that the H_2_, LiH, and FrH series (made from monovalent s-block elements) should exhibit some easily explicable trend. Unfortunately, *p*_AB_ fails to deliver a qualitatively convincing explanation in this case.

One may clearly see in Fig. 2 that part of the problem is that in the FrH molecule, the difference in sizes of two atoms and the very different *V*_i_/*R*_i_ values lead to enormous penetration of the vdW region of H, but not of Fr. A single index has a problem with grasping this complexity and the resulting consequences.

One will run into similar difficulties while trying to describe the very complex chemical bonding present in diactinide molecules; while the *p*_AB_ values for U_2_ and U_2_H_2_ are immense, some 200%,^12^ it is not exactly clear what these values mean and how they may be correlated with other important molecular parameters.

Summarizing, the chemical bond is a very complex object. It stems from a balance of weak and strong forces, some of which are attractive but many of which are repulsive; some electronic density is located directly between nuclei to decrease their repulsion (sigma bonds); however, some may be located in the regions departing from the straight line that connects the nuclei (pi, delta and phi bonds), or even far away from the atomic cores (as happens in electrides, for example). Charge transfer may occur. Excited states have a totally different distribution of electronic density than the ground states and usually longer bonds than the ground states, but fascinating exceptions are observed (take C_2_ or Be_2_, for example). Transition states of chemical reactions are characterized by complex multicenter bonding, where none of the key bonds of the reaction center are similar to what we are used to by studying the typical ground states. Last but not least, unusual bonding may be present for systems where substantial charge transfer takes place at appreciable distance, well above the sum of two vdW radii.^16^

The strength of the new approach by Alvarez and Echeverría^12^ is in its simplicity; any chemical bond may be analyzed in this way and reduced to a single number, which is obviously very useful and allows for drawing many meaningful (and even semi-quantitative) comparisons. Unfortunately, the weakness of this approach stems from the same source. As history teaches us, the paths of science are not straightforward. It remains to be seen how far the new index will bring us, and which important properties it can be correlated with, at least in the families of closely related chemical species. The strength of science is, *inter alia*, in the diversity of complementary ideas it can produce.

## Data availability

Data sharing is not applicable to this article as no datasets were generated or analyzed.

## Author contributions

W. Grochala wrote this article.

## Conflicts of interest

There are no conflicts of interest.

## Supplementary Material
